# The performance ceiling: why clinical data is insufficient for precision prognosis in concussion

**DOI:** 10.3389/fneur.2026.1801044

**Published:** 2026-05-04

**Authors:** Zach Napora, Owen Griffith, Semyon Slobounov

**Affiliations:** Sport Concussion Research and Service Laboratory, Department of Kinesiology, The Pennsylvania State University, University Park, PA, United States

**Keywords:** concussion, machine learning (ML), mTBI (mild traumatic brain injury), precision medicine, TBI - traumatic brain injury

## Abstract

The transition to active rehabilitation in concussion care requires precise tools to identify patients at risk of persistent post-concussive symptoms (PPCS). While machine learning (ML) offers the potential to personalize prognosis, current models relying on clinical history and subjective symptom reporting (e.g., SCAT5) have failed to demonstrate significant performance gains over the last decade. This perspective article argues that clinical prognostic models have reached a performance ceiling of approximately 0.85 Area Under the Curve (AUC). By reviewing key studies from 2016 to 2025, we demonstrate that increasing algorithmic complexity—from logistic regression to deep learning—yields diminishing returns when applied to subjective inputs. In contrast, models incorporating physiological data, such as neuroimaging or fluid biomarkers, consistently break this ceiling, achieving AUCs exceeding 0.95. We conclude that better mathematics cannot correct for missing biological signal, and that the advancement of precision medicine in neurotrauma requires a fundamental shift toward multimodal, biological data integration.

## 1 Introduction

The treatment of concussions has evolved significantly over the last couple of decades. The “rest is the best” approach has shifted toward active rehabilitation. Most patients functionally recover in about 4 weeks (1). However, a smaller subset of patients experiences prolonged symptoms, taking months and years to recover (2). While some risk factors of developing persistent post-concussive symptoms (PPCS) are understood, such as a history of migraines, pre-injury mental health history, and acute symptom severity, there are no widespread tools to prognosticate individual cases (3–5). This limits the application of targeted interventions in the acute phase of injury, such as selective brain cooling or vestibular therapy, which have the potential to lead to quicker symptom resolution (6, 7). In the last ten years, advances in machine learning (ML) and artificial intelligence (AI) have guided attempts to leverage large datasets of concussion patient information to develop predictive models of PPCS. Current prognosis of individual concussion recovery relies on clinical data, including the SCAT5 symptom data, demographic data, and history of migraines (8, 9). However, while ML and AI can optimize these models’ predictive power, there is a performance ceiling when using clinical data. Clinical models hit a performance ceiling at ~0.85 area under the curve (AUC). To break this, we must shift from patient self-reported data to multimodal models that incorporate objective multimodal biomarkers.

## 2 Discussion

### 2.1 The clinical performance ceiling

**Figure 1:**
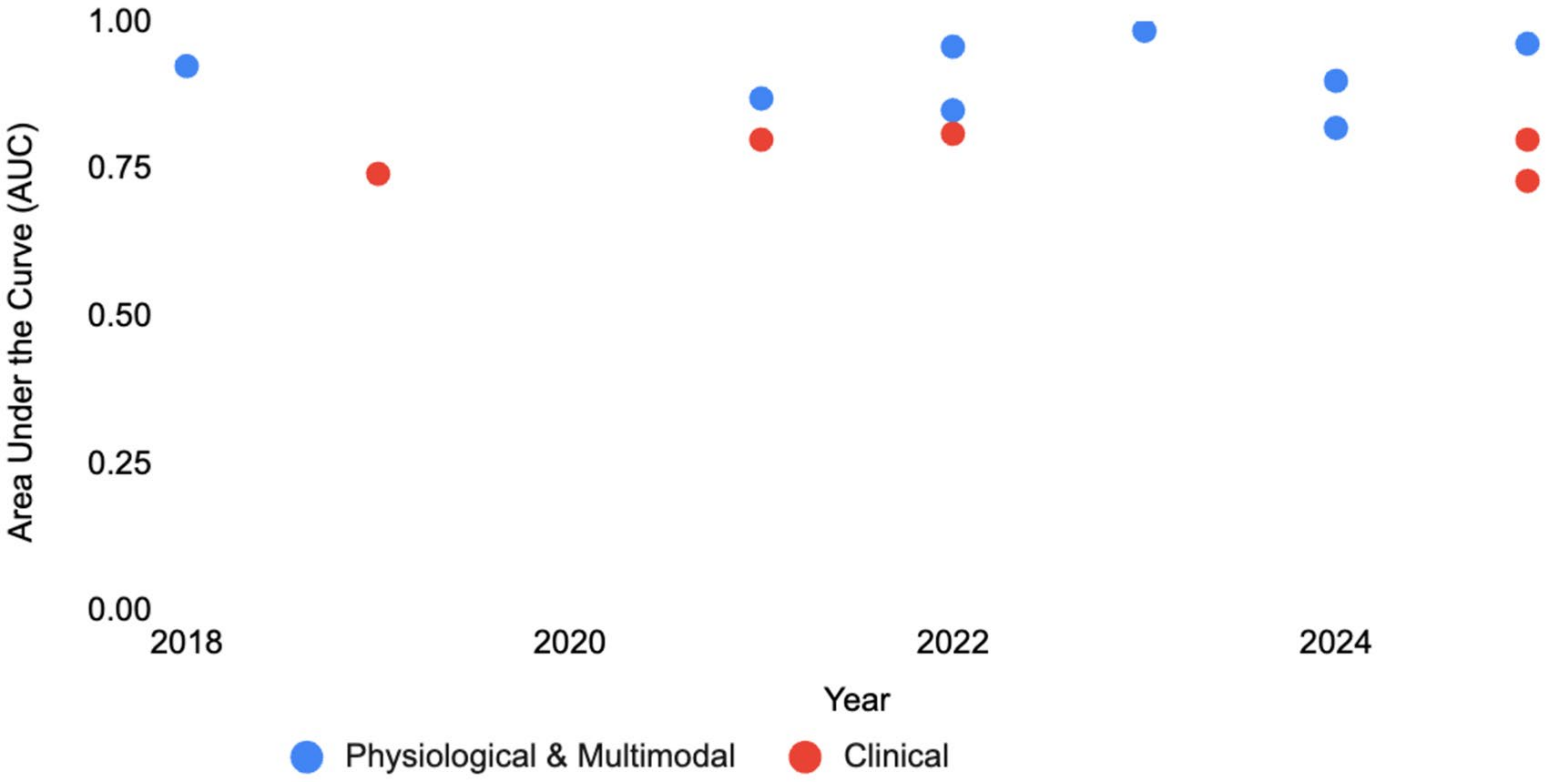
The ‘performance ceiling’ in concussion prognosis (2018–2025). A comparison of prognostic accuracy, as measured by the Area Under the Curve (AUC), for models predicting symptom resolution or functional recovery. Clinical models (Red) utilizing symptoms and demographics have plateaued between 0.74–0.81 AUC over the last six years, despite the application of advanced machine learning. In contrast, Multimodal models (Blue) integrating objective neuroimaging (MEG, MRI) or physiological biomarkers consistently exceed the 0.90 threshold, with recent fusion models achieving near-perfect classification (>0.95). The gap illustrates that predictive precision is currently limited by data source rather than algorithmic complexity.

Current risk stratification for persistent post-concussion symptoms (PPCS) relies heavily on self-reported symptom checklists and subjective clinical assessments, which have reached a predictive performance ceiling of AUC ~ 0.85 (Figure 1) (10, 11). Studies using advanced machine learning on clinical variables consistently report area under the curve (AUC) values between 0.65 and 0.81 (11, 12). For instance, even when utilizing specialized tools like the Vestibular Ocular Motor Screening (VOMS) and cognitive performance measures, clinical models for pediatric athletes peak at an AUC of 0.81 (11). In adolescent female cohorts, clinical-only models using Random Forest and Logistic Regression have achieved modest results with AUCs of 0.73 and 0.70, respectively (13). Furthermore, large-scale studies on sports-related concussion recovery using psychological and symptomatic factors report an AUC of 0.80, reinforcing a persistent plateau despite the use of sophisticated algorithms like CatBoost (14).

This plateau is further evidenced by the fact that emergency physicians’ diagnostic accuracy for predicting 3-month recovery based on clinical presentation is frequently no better than chance (15). Traditional clinical prediction tools face logistical difficulties in clinical settings and are less than 70% accurate (16). A primary driver of this ceiling is the inherent subjectivity of self-report measures, which are prone to the “good-old-days” bias, where patients tend to underestimate their pre-injury problems after a traumatic event (17). Additionally, common symptoms such as headache, fatigue, and dizziness are not specific to concussion and are frequently reported across healthy and injured populations alike (12, 17). Because subjective symptom reports can be manipulated by individuals seeking to expedite or delay return to activity, relying solely on these metrics creates significant uncertainty in clinical decision-making (16). Consequently, the diverse behavioral symptomology resulting from unique mechanisms of injury makes it notoriously difficult to predict recovery paths using observation alone (18). While clinical-only models frequently encounter a performance plateau when forecasting physical return-to-play, they demonstrate a significantly higher predictive ceiling for mental health sequelae (19, 20). This indicates that the subjective data and longitudinal history typically found in Electronic Health Records (EHR) may be inherently better suited. The most effective and accurate non-advanced imaging models utilized either neurocognitive data, longitudinal symptom inventories, psychological inventories, or vestibular/oculomotor screening alongside standard clinical data, strengthening the case for incorporating diverse data types for capturing psychological trajectories rather than for diagnosing acute physiological recovery (11, 20–22).

### 2.2 The multimodal breakthrough

While clinical and self-report models appear bound by an upper predictive limit, the integration of objective physiological biomarkers has demonstrated the capacity to shatter this performance ceiling, consistently achieving AUC values exceeding 0.90 (Figure 1) (23–25). By shifting the predictive focus from phenomenological observations to the underlying biological state of the brain, researchers have unlocked a higher tier of diagnostic and prognostic precision that is necessary for individualized care (Table 1).

**Table 1:** Summary of machine learning models for concussion prognosis categorized by data modality.

Author (Year)	Modality category	Input data	Algorithm	Outcome	Performance (AUC/Acc)
Clinical & symptom-based models					
Shafiei et al. (2016) (20)	Clinical	14 variables (Trauma history, substance use)	Neural Network (ANN)	Psychological Symptoms (6 mo)	AUC: 0.87
Nademi et al. (2018) (19)	Clinical	Demographics, History	Nonparametric Models	Psychological Symptoms	AUC: 0.86
Bergeron et al. (2019) (12)	Clinical	SCAT5 Symptom Scores	Machine Learning (Various)	Symptom Resolution	AUC: 0.74
Chu et al. (2022) (11)	Clinical	VOMS, King-Devick, Risk factors	CatBoost	Recovery Time	AUC: 0.78–0.84
Dabek et al. (2022) (32)	Clinical	EHR data, Demographics, Military Rank	Neural Networks, SVM	Mental Health Conditions	AUC: 0.82
Mao et al. (2025) (30)	Clinical	Daily Headache Diary (Longitudinal)	Partial Least Squares and Logistic Regression	Headache Trajectories	Accuracy: 0.80–0.84
Peng et al. (2025) (22)	Clinical	EHR data, SDoH, Pre-existing diagnoses	BiLSTM (Deep Learning)	Mental Health Diagnosis	AUC: 0.89
Bunt et al. (2025) (13)	Clinical	SCAT5, Demographics	Logistic Regression and Random Forest	Persisting symptoms	AUC: 0.70–0.73
Hellstrøm et al. (2017) (28)	Clinical	MRI Morphometry, Injury Data	Support Vector Regression	12-Month Outcome (GOSE)	r = 0.55
Thomas and Arnett (2025) (21)	Clinical	Demographics, injury characteristics (LOC/Amnesia), SCAT-3 symptom clusters, BSI-18 psychosocial scores, and ImPACT neurocognitive composites	Random Forest Classification	Prolonged Recovery (>28 days to return-to-play)	AUC: 0.85; Acc: 89.04%
Physiological & multimodal models					
Le Sage et al. (2022) (15)	Clinical (with CT)	Age, Sex, History, RPQ Scores	Logistic Regression	PPCS at 90 Days	AUC: 0.85
Hellstrøm et al. (2017) (28)	Multimodal	MRI Morphometry, Injury Data	Support Vector Regression	12-Month Outcome (GOSE)	r = 0.45
Jacquin et al. (2018) (24)	Multimodal	QEEG, Vestibular, Neurocognitive	Genetic Algorithm (GA)	Prolonged Recovery (>14 days)	AUC: 0.93
Fleck et al. (2021) (18)	Multimodal	DTI measures, Volumetric MRI	Genetic Fuzzy Trees	Symptom Recovery (1 week)	Accuracy: 0.62
Fedorchak et al. (2021) (16)	Biomarker	Salivary RNA, Balance, Cognition	Machine Learning	Symptom Duration (>21 days)	AUC: 0.86
Chen et al. (2022) (26)	Multimodal	fMRI (N-back), Neuropsych evaluation	SVM	Working Memory Decline	AUC: 0.96
Huang et al. (2023) (23)	Imaging	rs-MEG Source Magnitude Imaging	Machine Learning	Symptom Recovery	AUC: 0.99
Bertò et al. (2024) (10)	Imaging	DTI (White Matter Tracts)	Logistic Regression	Persisting Symptoms	AUC: 0.90
Cade and Turnbull (2024) (33)	Physiological	Computerized Eye Tracking	XGBoost (xgbDART)	mTBI Classification	AUC: 0.82
Yates et al. (2025) (25)	Multimodal	MRI Reports, SCAT5, Demographics	Random Forest	Games Missed (>5)	AUC: 0.96

AUC = Area Under the Curve; Acc = Accuracy; ANN = Artificial Neural Network; SVM = Support Vector Machine; BiLSTM = Bidirectional Long Short-Term Memory; DTI = Diffusion Tensor Imaging; fMRI = Functional Magnetic Resonance Imaging; MEG = Magnetoencephalography; QEEG = Quantitative Electroencephalography; SDoH = Social Determinants of Health; PPCS = Persistent Post-Concussion Symptoms.

The most significant leap in predictive accuracy has been achieved through resting-state magnetoencephalography (rs-MEG). Unlike clinical assessments that rely on a patient’s ability to perceive and report dysfunction, rs-MEG directly measures spontaneous neuronal activity with high spatial and temporal resolution (23). Huang et al. (23) developed a machine learning algorithm that integrated rs-MEG source imaging markers across both delta (1–4 Hz) and gamma (30–80 Hz) frequency bands. This combined model predicted pediatric mTBI cases against orthopedic controls with a sensitivity of 95.5% and a specificity of 90.2%, achieving a peak AUC of 0.985 (23). This represents the highest reported performance in the literature and underscores that regional hyperactivity—particularly in the frontal lobe poles—serves as a definitive neural injury signature that clinical checklists cannot capture (23).

Objective measurements of the brain’s structural framework have also proven superior to clinical data modeling. Utilizing diffusion-weighted MRI (dMRI) and tractography, researchers can identify microstructural disruptions in white matter that correlate with recovery time (10, 25). Bertò et al. (10) leveraged the mean Fractional Anisotropy (FA) of 16 statistically significant white matter tracts to develop a prognostic model for collegiate athletes. Their Logistic Regression binary classifier achieved an AUC of 0.90 and, notably, a sensitivity of 1.0, meaning the model identified every athlete at risk for persistent symptoms without a single false negative (10). Similarly, Yates et al. (25) demonstrated that integrating injury history with automated MRI reports identifying white matter hyperintensities yielded a composite model accuracy of 94.6% and an AUC of 0.963. These findings suggest that the physical state of white matter tracts, such as the inferior fronto-occipital fasciculus, provides a more reliable prognostic signal than symptom-based resolution (10, 25).

The breakthrough into high-precision forecasting also extends to the prediction of specific cognitive sequelae. While traditional neuropsychological evaluations often fail to predict long-term changes, functional MRI (fMRI) biomarkers measured during the acute phase have shown high sensitivity to future working memory (WM) deficits (26). Chen et al. (26) utilized machine learning to analyze WM task-induced activation and deactivation maps at baseline. Their model achieved an AUC of 0.958 in identifying patients whose working memory ability at one year post-injury would be worse than their pre-injury baseline (26). This suggests that early functional connectivity patterns—specifically the imbalance in communication between task-positive and task-negative regions—can forecast a chronic course that clinical inventories might miss during transient periods of symptomatic recovery (26, 27).

The breakthrough in performance is best exemplified by models that synthesize multiple physiological modes into a single index. Jacquin et al. (24) developed an enhanced multimodal Brain Function Index (eBFI) that combined quantitative EEG (QEEG), neurocognitive throughput, and clinical vestibular measures. This integrated approach achieved an AUC of 0.925, successfully separating controls from concussed subjects with prolonged recovery (24). Even in cases where individual biological markers do not break the 0.90 threshold, such as salivary ncRNA, they still provide significant additive utility. Fedorchak et al. (16) showed that adding a panel of 16 salivary RNAs to a validated clinical risk score boosted the predictive accuracy from a modest 0.73 to a robust 0.86 AUC.

While multimodal machine learning models typically demonstrate superior prognostic performance by integrating objective physiological data, the findings of Hellstrøm et al. (28) serve as a critical methodological baseline illustrating that the specific type of imaging data used determines whether a model can exceed the established clinical performance ceiling (10, 23). In their study, a clinical-only model (r = 0.55) actually outperformed a combined clinical and imaging model (r = 0.45) because macrostructural MRI cortical morphometry and subcortical volumes performed at a chance level (r = 0.03), offering no added predictive value over demographic and clinical features (28). This result highlights that traditional structural imaging is often insensitive to the subtle pathophysiological changes of mild traumatic brain injury, which contributes to clinical models plateauing at AUC values between 0.65 and 0.81 (11, 12). To move beyond this plateau, the sources suggest shifting toward microstructural or functional imaging modalities; for instance, models utilizing diffusion-weighted MRI to analyze white matter tracts or resting-state magnetoencephalography (rs-MEG) have consistently surpassed clinical-only metrics, achieving peak AUCs ranging from 0.90 to 0.985 (10, 23). Ultimately, the Hellstrøm results highlight that while multimodal integration is vital for precision medicine, true predictive superiority is only realized when the selected biomarkers move beyond phenomenological observation to capture the specific biological or electrical signatures of the injury (18, 23, 28).

### 2.3 The diminishing returns of algorithmic complexity

The evolution of machine learning (ML) architectures in concussion research marks a shift from traditional linear statistics toward non-linear systems capable of managing the inherent complexity and heterogeneity of brain injuries (12). These diverse architectures—ranging from supervised classifiers to sequential deep learning and unsupervised pattern discovery—have fundamentally changed how researchers identify risk factors and predict recovery trajectories (29, 30). Supervised algorithms, particularly Support Vector Machines (SVM) and Random Forest (RF), have become the primary tools for clinical risk stratification, and are among the first algorithms used for the classification of concussion (31). SVMs have proven exceptionally powerful when applied to high-dimensional physiological data; for example, an SVM model utilizing Recursive Feature Elimination on rs-MEG source magnitude imaging achieved a near-perfect 0.985 AUC in distinguishing pediatric mTBI from controls (23). Similarly, SVM architectures have successfully predicted working memory decline at one year with over 83% accuracy by processing task-based fMRI activation maps (26). Random Forest models provide additional impact through their ability to rank variable importance without overfitting (13). This architecture allowed researchers to identify that emotional symptom clusters and injury history are most predictive of significant time out of sport, achieving a composite model accuracy of 94.6% (25).

A significant limitation of early models was their “static” nature, providing a single forecast at the time of injury. To address the time-varying nature of recovery, researchers have implemented Sequential Learning architectures like CatBoost and Bidirectional Long Short-Term Memory (BiLSTM) networks. CatBoost has been instrumental in processing multi-dimensional categorical data from vestibular and ocular-motor screenings, producing more parsimonious models that outperform traditional human-driven regression (26). Furthermore, the application of BiLSTM networks to Electronic Medical Record (EMR) data has enabled real-time, visit-by-visit risk assessments (22). By embedding temporal information, these models can adjust a patient’s mental health prognosis as new clinical symptoms or subsequent injuries emerge during the 12 months following a concussion (22, 32).

While supervised models predict known outcomes, unsupervised learning architectures have been used to discover hidden recovery patterns that clinical definitions may miss. Using Locality-Sensitive Hashing (LSH) and autoencoders, researchers identified 11 distinct blood biomarker trajectories, revealing that specific rising patterns of GFAP and NF-L are associated with higher risks of loss of consciousness (29). Similarly, tensor decomposition has been used to subgroup patients into four unique headache evolution trajectories, allowing for the early identification of individuals whose symptoms will remain severe without intervention (30). The impact of these discovery-based models is a shift toward precision medicine, where treatments are tailored to a patient’s specific biological subtype rather than a broad mTBI diagnosis (29, 30).

Novel architectures such as Genetic Fuzzy Trees (GFT) and Genetic Algorithms (GA) offer a unique impact by modeling biological decisions using “if-then” linguistic rules (18). GFT systems are particularly robust against the uncertainty and small sample sizes common in neuroimaging data, outperforming six other ML methods in predicting symptomatic recovery based on DTI white matter measures (18). In parallel, Genetic Algorithms have been used to synthesize disparate data types—including QEEG, neurocognitive throughput, and vestibular signs—into a single multimodal Brain Function Index (24). These specialized systems demonstrate that while architecture is vital for handling data complexity, the most significant performance leaps occur when the algorithm is designed to integrate objective, multimodal physiological inputs (18, 24).

### 2.4 Toward scalable precision medicine

The current evidence suggests that concussion management is gradually transitioning from a reliance on subjective clinical assessment toward a precision medicine framework (15, 29). This shift appears to be necessitated by the inherent heterogeneity of mild traumatic brain injury, which varies significantly in its causation and recovery trajectory (29). While traditional clinical checklists remain the mainstay of practice, they may be reaching a predictive performance plateau that limits the ability of clinicians to accurately identify individuals at high risk for persistent disability (10, 11).

The integration of objective, multimodal physiological data provides a potential pathway to exceed this clinical ceiling. Advanced neuroimaging modalities have demonstrated the capacity to identify neural injury signatures that are often invisible to standard clinical screening (10, 23). Furthermore, the identification of biological endophenotypes through unsupervised learning may enable more targeted and individualized interventions, potentially reducing the risk of persistent symptoms (13, 29, 30). Ultimately, the shift toward a data-driven precision medicine approach may significantly enhance the accuracy of concussion forecasting compared to unassisted clinical judgment and raise the clinical relevance of advanced imaging techniques following concussive injury (22, 32).

## Data Availability

The original contributions presented in the study are included in the article/supplementary material, further inquiries can be directed to the corresponding author.
